# Supplementary material for: An optogenetics device with smartphone video capture to introduce neurotechnology and systems neuroscience to high school students
Source: PLoS One. 2022 May 6;17(5):e0267834. doi: 10.1371/journal.pone.0267834 (PMC9075642; doi:10.1371/journal.pone.0267834)
Supplement: S1 Fig — (PDF) [file pone.0267834.s001.pdf]

Front View

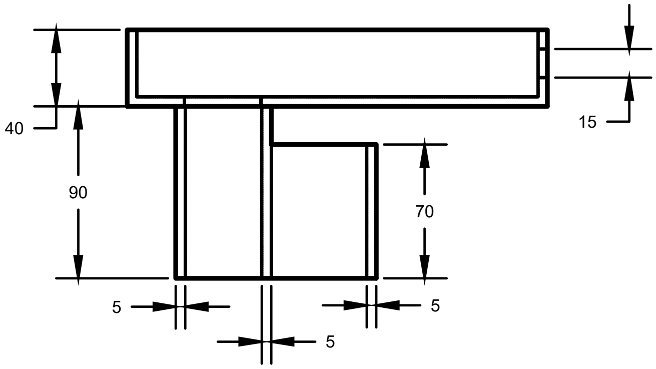

Left View

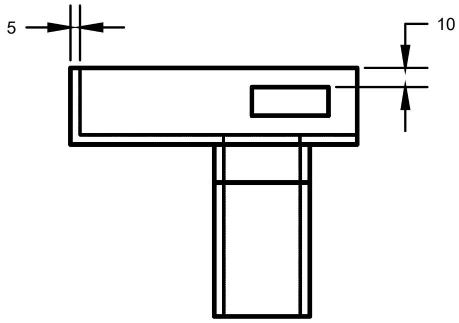

Top View

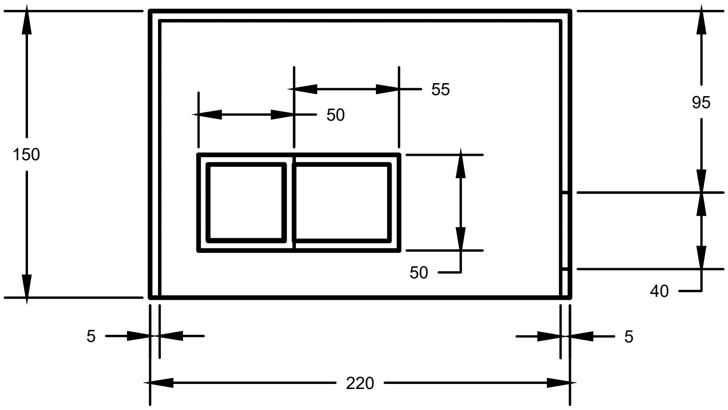

3D Model

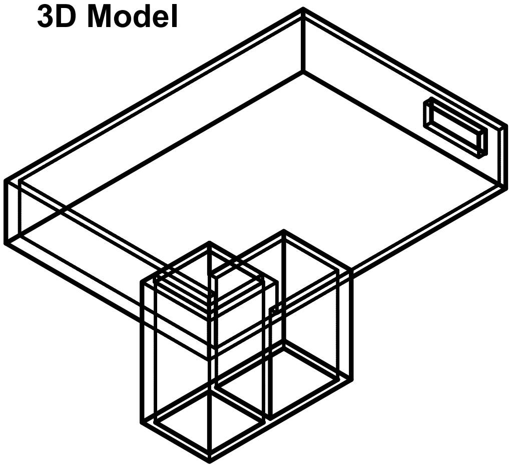

Stereoscope Camera Mount

Materials: Cardboard

All dimensions are in millimeter units
