# Supplementary figures and images for: An optogenetics device with smartphone video capture to introduce neurotechnology and systems neuroscience to high school students
Source: PLoS One. 2022 May 6;17(5):e0267834. doi: 10.1371/journal.pone.0267834 (PMC9075642; doi:10.1371/journal.pone.0267834)

Circuit Diagram

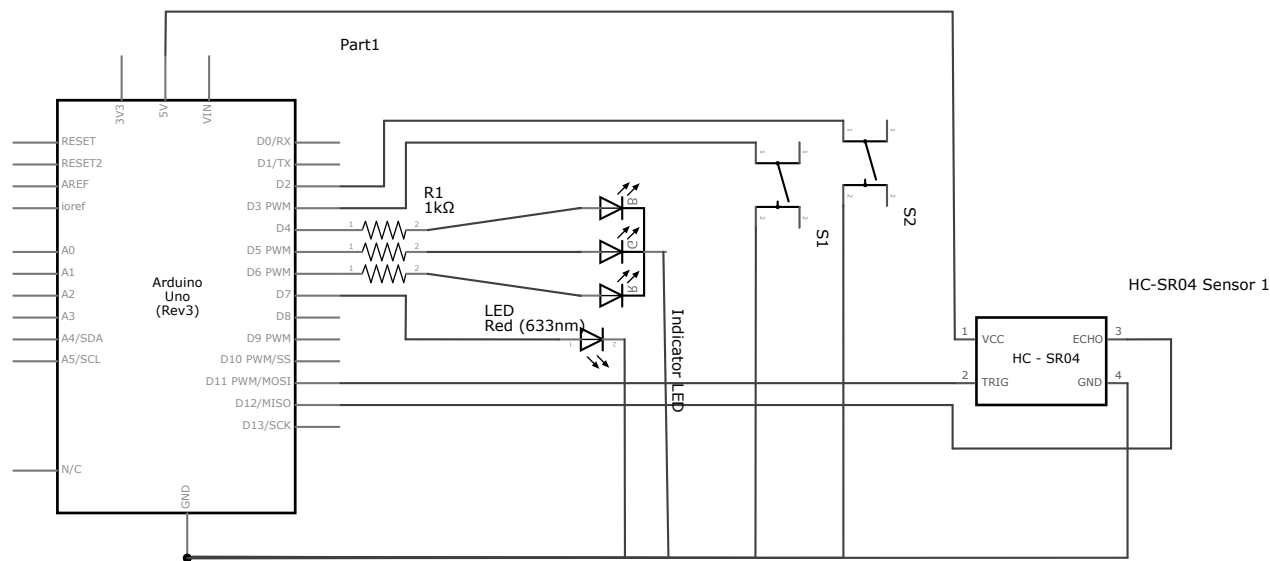

Connectivity Diagram

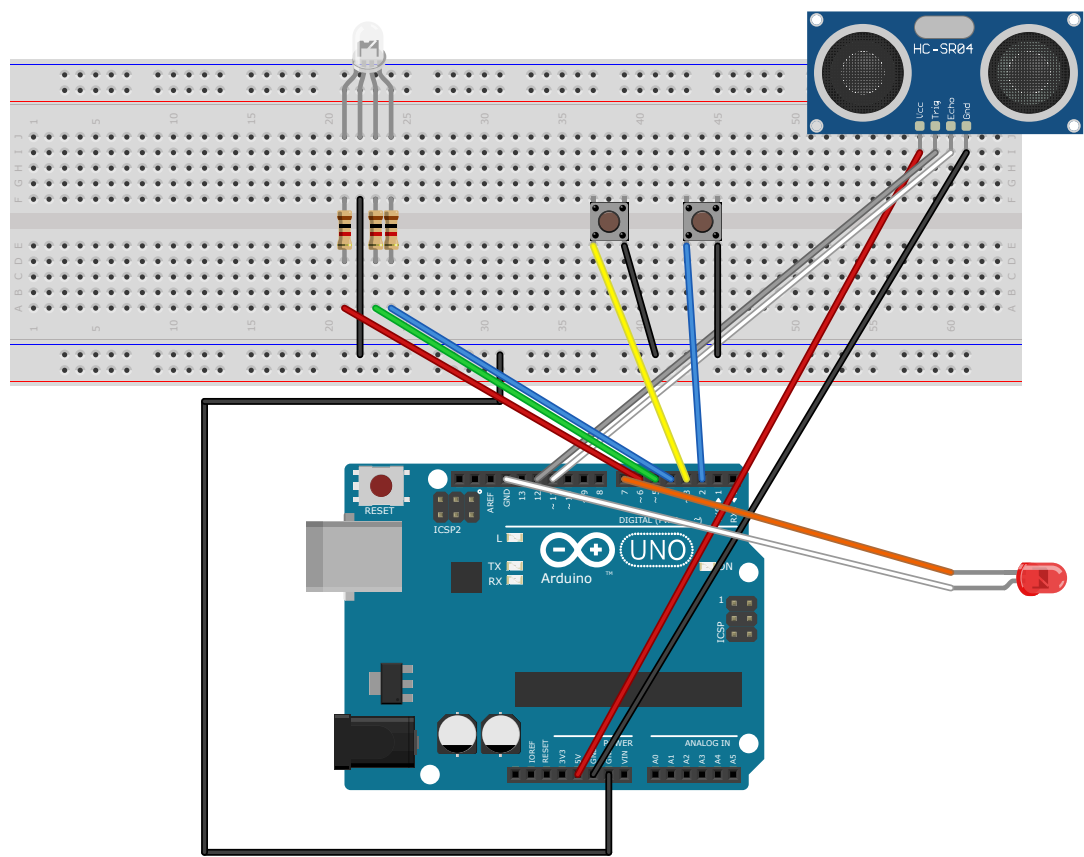

Supplement: S2 Fig — (PDF) [file pone.0267834.s002.pdf]
