# Supplementary material for: An optogenetics device with smartphone video capture to introduce neurotechnology and systems neuroscience to high school students
Source: PLoS One. 2022 May 6;17(5):e0267834. doi: 10.1371/journal.pone.0267834 (PMC9075642; doi:10.1371/journal.pone.0267834)
Supplement: S1 Table — (PDF) [file pone.0267834.s003.pdf]

S1 Table. Materials with purchasing information.

**Material**	**Approximate Cost**	**Link**
Transgenic Flies	~$22.50 per stock	https://bdsc.indiana.edu/stocks/index.html
Fly Food	Price varies depending on recipe used	https://bdsc.indiana.edu/information/recipes/bloomfood.html
All-trans-retinal	~$189.00 for 500mg (makes ~ 1000 vials of food)	https://www.sigmaaldrich.com/US/en/product/sigma/r2500
Tungsten Wire - 0.1mm	~$13.99	https://www.amazon.com/RINGGLO-Tungsten-0-01mm-1mm-Machinery-Manufacturing/dp/B09J2CNVMX/ref=sr_1_2?crid=5P1KIYSJNV01&keywords=tungsten%2Bwire%2B.1mm&qid=1643207026&sprefix=tungsten%2Bwire%2B.1mm%2Caps%2C46&sr=8-2&th=1&psc=1
Liquid Plastic Adhesive Kit	~$19.95	https://www.amazon.com/FlashFix-Plastic-Adhesive-Seconds-Complete/dp/B099TFPPF4/ref=sr_1_2_sspa?crid=ALVAUD574WK9&keywords=uv+glue+kit+with+light&qid=1643207176&sprefix=UV+glue+%2Caps%2C78&sr=8-2-spons&psc=1&spLa=ZW5jcnlwdGVkUXVhbGlmaWVyPUEyOVdHS0lQNDhOUE01JmVuY3J5cHRlZElkPUEwNTE1MzMyMlFCTUdUOEdDNEtRSyZlbmNyeXB0ZWRBZElkPUEwOTc3OTcxMTNNNVRLUUtSQkYxSyZ3aWRnZXROYW1lPXNwX2F0ZiZhY3Rpb249Y2xpY2tSZWRpcmVjdCZkb05vdExvZ0NsaWNrPXRydWU=
ELEGOO UNO Project Super StarterKit	~$38.99	https://www.amazon.com/ELEGOO-Project-Tutorial-Controller-Projects/dp/B01D8KOZF4
LUXEON Rebel 627nm LED, LuxeonStar	~$7.50 each	https://www.luxeonstar.com/red-627nm-10mm-square-led-102lm
Modeling Clay	~$6.95	https://www.amazon.com/Claytoon-228051-Modeling-Assorted-Colors/dp/B004O7HLBQ/ref=sr_1_5?keywords=claytoon+modeling+clay&qid=1643207597&sprefix=claytoon+%2Caps%2C63&sr=8-5
Double-sided tape	~$12.99	https://www.amazon.com/EZlifego-Multipurpose-Removable-Transparent-Household/dp/B07VNSXY31/ref=sr_1_5?keywords=double-sided+tape&qid=1643207641&sprefix=double-sided%2Caps%2C67&sr=8-5
Cardboard	N/A	N/A
Stereoscope	N/A	N/A
