# Supplementary material for: An optogenetics device with smartphone video capture to introduce neurotechnology and systems neuroscience to high school students
Source: PLoS One. 2022 May 6;17(5):e0267834. doi: 10.1371/journal.pone.0267834 (PMC9075642; doi:10.1371/journal.pone.0267834)
Supplement: Attachment — Submitted filename: 2021_optogenetics_workshop_plos_one.pdf [file pone.0267834.s007.pdf]

I would like to thank the authors for putting this manuscript together and also for creating such a low cost device for high school workshops. They describe a system that is quite affordable ~40 dollars for most parts on Amazon, and serves to perform optogenetic experiments in a classroom setting, which is quite nice. I hope they see my comments as positive criticism and a way to make their manuscript even more robust. I am happy to discuss any comments further and provide clarification where necessary.

I like the rationale put in the introduction and how it breaks down things step by step. The one shortcome, is that it fails to mention other open source optogenetics systems and make a brief comparison with the one described here. A brief pubmed search brought up these resources (1, 2, 3, 4), maybe they are pertinent for the paper/comparison? (from what I see, the device described here is the most affordable one).

Another general point is that for this system to work, users need to have access to a smartphone (I understand that we normally think most people have one, but this is not necessarily true for high school students outside US, Europe, and certain regions in Asia), and a stereoscope (this is def. Not always the case in all high schools). If the authors could add a couple of words on alternative ways to still perform the experiments using other resources, I think this would help readers interested in bringing this to their classrooms.

Line 67 Could the authors expand a bit the phrase relating to optogenetics being predicted to be less invasive for human brain stimulation? It is less invasive in comparison to what, and in what sense? My rationale is that for human brain stimulation you would need to genetically alter brain regions to express specific proteins and depending on areas to be stimulated, one would still need to insert a fibre/fibres.. Which makes “invasive” a very relative term?

Line 204 is the phone app only available for Iphone? If this is the case, please add alternatives for Android phones. If that is not available, please state it in the text.

Line 244 Please add a link to the summer academy program where the workshops take place

Please add links to the materials needed. I know you write the name of the kits and the red LED, but adding the links will help readers procure them. If they have Amazon available, this will make ordering faster, and if not, they will be able to at least see what they look like and find similar products.

If possible, add a pdf version (or other available file format) of the protocols.io page to your github repository, so that there is at least one place all needed protocol information is available.

Giant Fiber abbreviation is introduced twice, once in line 216 and once in line 311. Please correct.

I like the automatic triggering of the arduino with the ultrasound sensor, but it would be useful to see more details on why this was created in the “Controller” section of the paper. What level of synchronisation was achieved? In other words, using this system, would the optogenetic protocol always start at a specific time interval after the video started? What was the error? I am trying to understand if this sync system is crucial for this setup, or if it is more of a “nice to have”.

On your protocols.io description, please add photos or a video showing step 13, as this is a crucial step of the preparation.

Here are a couple of minor typos:

Line 316 two spaces after the full stop
Line 318 space between word and full stop
Line 339 “labled”

Figure 4. I get the effect of the workshops is significant, but I would like to see the raw data plotted here. This would allow for a better insight on how the student’s prior level of knowledge is spread, and how less spread it becomes after the workshops. If I understood correctly you have ~45 students in total, and there is no obvious reason (at least not described on the text) to split the data into workshop groups. So it would be possible to simply plot all the data points, and indicate where means and percentiles are (something similar to this)
