# Supplementary material for: An optogenetics device with smartphone video capture to introduce neurotechnology and systems neuroscience to high school students
Source: PLoS One. 2022 May 6;17(5):e0267834. doi: 10.1371/journal.pone.0267834 (PMC9075642; doi:10.1371/journal.pone.0267834)
Supplement: Attachment — Submitted filename: Response to Reviewers.pdf [file pone.0267834.s008.pdf]

Dear Academic Editor Dr. Matthieu Louis,

Thank you for this opportunity to resubmit our article. We also appreciate the feedback from the reviewers that has enabled us to improve clarity and to recognize the work of others who have contributed to this underserved area. Our comments to the reviewers and changes to the manuscript are provided below. Line numbers correspond to the "Revised Manuscript with Track Changes" version of the manuscript.

Major Request Summary:

I. Provide a better description of the existing literature and acknowledge previous work done to bring Drosophila neuro-engineering to high schools. Add missing references. Soften unnecessary or unjustified claims about novelty (requested).
   - *We apologize for missing references and have added previous work and softened claims as requested in the abstract and introduction (see below).*

II. Complete missing information related to the methodology (requested).
   - *We have added the missing information as requested in the methods section (see below).*

III. Add a bill of material (table) with the reagents and providers (requested).
   - *We have added a bill of materials as a supplemental table with links to where to purchase the materials as requested (see below).*

IV. Discuss alternatives to the use of a smartphone and stereoscope (recommended).
   - *We have added potential alternative solutions to the discussion section as requested (see below).*

V. Ensure that the cell-phone application works for both iPhone and Android phones (strongly recommended).
   - *Although our original app was only compatible with iPhones, we have found an Android app that is sufficient for students to capture and annotate fly videos. Additionally, major gains in cell phone video technology (some Android phones are capable of 960fps at 720p) are making an app unnecessary. We now comment on this in the methods and discussion section (see below).*

Response to Reviewer 1:

1. **<u>Improvement of layout (arrow-based representations) and information represented in the figures (strong recommendation).</u>** Fig. 1E: The arrows are confusing if not misleading. For example, Ultrasound module has an arrow to the status indication. But the Ultrasound module does not control it. Another example is the Board LED module. This has an arrow to the Camera mount. But the board LED does not control or manipulate the camera mount. Also, what are the switch module and the stereoscope LED module (although I can guess)? They are not explained in anywhere in the text. I strongly suggest revising this figure and clearly explaining every component and arrows.

   We appreciate the feedback for this figure and have attempted to make this figure clearer. All modules in this figure have been renamed to match those seen in the methods section to improve consistency. To remedy the incorrect information flow in Figure 1E, status LED module has been moved into the "Arduino Development Board" region of the figure. Additionally, information is now shown traveling between the control program and the motion sensor module to accurately depict the interactions of this

module. A new box was created to make a "Stereoscope Camera Mount" region of the figure and now shows the smartphone and motion sensor module housed by the stereoscope mount. The optogenetic stimulation LED module was moved out of the "Arduino Development Board" region of the figure to distinguish its placement relative to the other modules and the Arduino. Finally, the following description of every element in this figure has been added to the Figure 1E description (Lines 260-265).

"(**E**) Component diagram for the device. The Arduino Development Board controls the Connected Switch Module, Status LED Module, Board LED Module, Optogenetic Stimulation LED Module, and Motion Sensor Module necessary for this experiment. Modules depicted with double-sided arrows communicate to and from the Arduino for proper module function. The Stereoscope Camera Mount houses both the Motion Sensor Module and a user's smartphone."

2. **Improvement of the statistics and potential increase of sample sizes (Figure 4) or a better justification of the appropriateness of the statical tests used (requested).** In Figure 4, performing statistical test on sample size 3 is not well justified. I suggest collecting at least 5 samples (desirably 10 samples or more) for any statistical tests. If this is not feasible, consider other ways of testing the effectiveness. (E.g., instead of using the average point of each session, individual students' points can be tested using 2-way Anova for each question, which should provide more believable statistics. Or even three-way Anova may be considered for the entire dataset.)

   The data were collected from 47 students across the three workshops so we now use as N individual students, as suggested. We have also applied a Wilcoxon signed rank test to each question (Figure 4 and Figure 4 caption, Lines 928-932).

   "**Fig. 4. Workshop assessment outcomes.** A total of 47 students distributed over three workshops were evaluated on four concepts, both before and after the workshop. Gray connected boxes indicate individual student scores and red squares indicate averages (Wilcoxon signed rank test, ***=p<<0.001). Abbreviations: quiz administered before the workshop (B); quiz administered after the workshop (A)."

3. **Discuss how high school students can obtain trans-retinal. Discuss ways to mitigate potential barrier in the use of the tool in an educational context (strong recommendation).** In Discussion: On a side note, I believe that fly genetics and optogenetics can bring a huge impact on K-12 education. There is a potential to renovate the biology curriculum in K-12 schools. However, the biggest barrier to fly optogenetic experiments in general public is to find the all-trans-retinal (ATR) food, which requires purchasing ATR powder, dissolving it in ethanol, and keeping it in a low temperature until mixed in the regular food. All of these steps are close to impossible for high school students to do by themselves. In most cases, such food can only be obtained via fly labs in a nearby university. As a result, most students cannot perform exciting optogenetic experiments at home for an extended period time, which is essential to develop scientific insights. Although this may be well beyond the scope of this study

and not necessary for this study, it would be appreciated if authors share any idea about, if any, or discuss how to mitigate this major barrier.

We agree that obtaining retinal is a barrier and have expanded upon this in the discussion (Lines 986-1040).

“At present, the workshop requires a partnership with a *Drosophila* research laboratory in order to obtain all-*trans*-retinal fly food, as there is no current commercial source. β-carotene has been used as an alternative to all-*trans*-retinal in Drosophila optogenetics experiments [49]. Adding carrot juice to fly food could therefore be a possible low-cost, easily accessible alternative but this has yet to be tested.”

4. I suggest including the actual light power of the 627 nm LEDs measured at 2 mm distance. Even though high school students may not need to know the details, the reader of the paper may want to know the technical specs of the system. Related, wouldn’t a regular red LED (not a high power LED) activate GF?

We have revised this information and moved it to a more appropriate earlier section of the methods after remeasuring our LEDs at 2mm (Lines 611-614). Reviewing power levels from our earlier publications using these same driver and CsChrimson lines, as well as from other labs using these same driver lines, reported values at the location of the fly range from (0.025 to 0.500 mW/mm^2). For CsChrimson activation in behaving flies using other genotypes, measured light intensities range from 0.04 to 0.4 mW/mm^2 (DeAngelis, B. D. et al., 2020; Mohamed, A. A. M. et al., 2019; Musso, P. Y. et al., 2019; Schretter, C. E. et al., 2020). As long as an LED is able to generate this same level of power at the correct location and wavelength, it should be appropriate. However, all other LEDs will need to be tested, so we prefer to keep in the text the exact LED type that worked for our experiments and for the high school student workshop.

" After a 1.5 second delay, the level of digital pin D7 was changed from LOW to HIGH for 300 milliseconds, causing a 300-millisecond activation stimulation from the red LED (measured to be 0.16 mW/mm^2 at the fly’s location, 2mm away from the LED).”

5. In the Introduction & Discussion: Low-cost devices to perform optogenetics or behavioral experiments with Drosophila have been developed in multiple labs. (Below are such examples.) These different approaches need to be compared and discussed. For example, the setup of the current manuscript is even more cost-effective and easier to implement, which can be emphasized. Or, the current setup can be implemented by tech-savvy high school students without the help of instructors.

   - The 100 Euro Lab: A 3D-printable open-source platform… PLOS Biology, Chagas et al., 2017
   - PiVR: An affordable and versatile closed-loop platform… PLOS Biology, Tadres and Louis, 2020

- An inexpensive, High-Precision, Modular Spherical Treadmill Setup… Frontiers in Behavioral Neuroscience, Loesche and Reiser, 2021

Thank you for the missing references and for highlighting novelty of our device. We have added these and additional references (included those suggested by the other reviewers), acknowledged their contributions, and highlighted remaining obstacles in the introduction (Lines 73:77, 93:97, and 111-113).

"In recognizing this need, there have been concerted outreach efforts from higher education, the nonprofit research sector, and the private sector to develop low-cost versions of cutting-edge devices that enable high school students to employ neurotechnologies and engage in hands-on activities to learn neuroscience concepts [8, 19-27]."

"To increase accessibility, recent efforts have succeeded in establishing significantly lower-cost optogenetics devices [19, 20, 22, 25]. However, some of these elegant solutions have been designed for undergraduate labs and/or require 3-D printing that may not exist at high schools with limited budgets."

"However, many low-cost optogenetics devices designed for high school students do not provide a solution for synchronized light activation and video capture [27, 37]."

6. Line 172-173: I assume that the turning on the 627nm LED is delayed for some amount of time (e.g., 3 seconds) to wait for the phone camera to start recording. Please clarify.

   This is correct and we apologize for leaving this important detail out of the original submission, we have revised and added the following to the "Optogenetic Stimulation (LED) Module" section (Lines 631-639).

   "After a 1.5 second delay, the level of digital pin D7 was changed from LOW to HIGH for 300 milliseconds, causing a 300-millisecond activation stimulation from the red LED (measured to be 0.16 mW/mm^2 at the fly's location, 2mm away from the LED). At the end of the LED stimulation, the device entered a 30 second refractory period. During this period, students would stop their video recording and the Optogenetic Stimulation LED could not be re-activated, preventing students from over-stimulating their fruit fly and providing enough time for CsChrimson to recover from deactivation [46]. The timing of the video capture and light stimulation enabled students to consistently collect fly behavior before, during and after the light stimulation."

7. The fly and the red LED are a bit small Fig. 1C compared to other figures in Fig. 1. You may want to enlarge it.

   The size of Fig 1C has been increased to match the other figures in Fig 1.

8. Fig. 1C: The fly is tethered at the tip of a wire. I suspect that the wire might be very wobbly. How do you stabilize it?

   To address this concern, we have reworded the word "Pin" in the description of Fig 1C (Lines 255-257) to "tungsten wire" to be clear what we're using. Additionally, we provide the diameter of the wire (Line 672-674). We find that 0.1 mm tungsten wires enable us to adjust the viewing angle of the fly without compromising stability during a recording, as they easily support the weight of the fly.

   "UV glue connects one end of a tungsten wire to the notum of the *Drosophila*. The other end of the tungsten wire is inserted into a piece of clay for positioning and stabilization."

   "Single anesthetized flies were then tethered to one end of a 0.1 mm tungsten wire with UV glue under a stereoscope, using a small paintbrush to position and restrain the fly. The other end of the tungsten wire was inserted into clay for stability and future positioning."

9. Line 119: typo?: It is consisted of

   We have edited this sentence to remove the awkward phrasing.

10. Using a cardboard to mount a phone is a great idea to reduce the cost because most phone mounts are at least $15. But the proposed design seems to work only with the Leica EZ scope. The phone mounting part and the part covering the eyepiece could be separated in the supplementary figure and you can offer a multiple designs or instructions to build for other brands of stereoscopes.

    Thank you for this suggestion, in the methods we have now added a section on how to adapt the Stereoscope Mount for other stereoscopes (Lines 514-520).

    "We designed our Stereoscope Camera Mount around the Leica EZ4 stereoscope, however the sleeves connecting the platform to the stereoscope eyepieces can be modified length and width wise to accommodate any stereoscope. After constructing the sleeves for the eyepiece, if further adjustments are necessary to ensure a snug fit, modeling clay can be inserted between the stereoscope and the sleeves to adjust the z-axis, or in between the sleeves and eyepieces to adjust the xy-axis. The platform dimensions should be compatible across stereoscopes and should be capable of supporting any smartphone."

11. Line 134: Wiring diagram uses a prototyping breadboard, but in Fig. 1, a shield is used. Although this is a very minor issue, it may be a good idea to clarify this for those who are not familiar with the Arduino ecosystem.

    This observation is correct, and we have added the following sentence (Line 259-260) to rectify this issue.

"The Arduino Development Board depicted in this figure has a shield that replaces the role of an ordinary protoboard."

12. Line 194: Anesthetization of flies on ice for 10 minutes may easily kill the fly. Authors may have used a method to prevent hemolymph from freezing. That is, the temperature may have been higher than 0 C. Please clarify. (E.g., A vial with fly may be place in an ice bucket for a couple of minutes, then can be transferred to a plate with a temperature at around 2 C.)

    We have tested flies kept on ice in a conical tube after 1 hour and found they are still able to recover and perform behaviors. We have added the conical tube information as a clarification in the text (Line 652).

    "*Drosophila* were anesthetized in a conical tube on ice for approximately 10 minutes."

13. Line 195: Was the gluing done under a microscope? Was the fly constrained in a sarcophagus?

    We edited this section of the text to clarify that gluing was done under a stereoscope. The fly was not constrained in a sarcophagus but we did use a paintbrush to position the fly during gluing. The fly was immobile due to prior anesthetization on ice (Lines 652-654).

    "Single anesthetized flies were then tethered to one end of a 0.1 mm tungsten wire with UV glue under a stereoscope, using a small paintbrush to position and restrain the fly."

14. Line 197: Why do you place the fly in a freezer box (below 0 C)? Or is it just a Styrofoam box without ice?

    We edited the text to clarify that flies can be placed in any covered box, as the purpose of the box is to avoid unintended light stimulating the fly (Lines 655-658).

    "Flies were then placed in covered boxes to recover in the darkness for more than five minutes prior to the start of experiments. Flies were kept in covered boxes between experiments to avoid unintended light stimulation."

15. Line 202, 264: Does not have to be an iPhone. Or does it?

    In validating the device and in running the workshop, we relied on iPhones. However, we have found a slow-motion simulation app for Android phones that is sufficient for behavior identification. We also mention that the newest Android phones can be used without an app (Lines 663-691).

    "The stereoscope was then used to focus the smartphone camera on the fly via a slow-motion video app (SloPro, https://apps.apple.com/us/app/slopro/id507232505). We chose the SloPro app

because it uses optic flow to simulate 1000 frames per second (fps) high speed video capture. This permits students to recognize the initiation or cessation of evoked behaviors that are quite rapid and difficult to observe by the human eye and not be limited by the highest frame rate of their smartphone camera. We also identified a slow-motion app for android phones (Slow Motion Video FX, https://m.apkpure.com/slow-motion-video-fx/com.mobile.bizo.slowmotion) that is sufficient for behavior annotations. We anticipate, with the continuous development in smartphone video technology (the newest iPhones and Android phones are capable of 240 fps at 1080p and 960 fps at 720p, respectively) a high speed "simulation" app will not be required in the near future."

16. Line 217: Placing the Kimwipe for the fly to hold (and its purpose) should be mentioned before this line.

    Thank you for this advice, we have added additional information about the purpose of the Kimwipe and the behavioral state of the fly (Lines 693-699) as it is important for reproducing these experiments.

    "Tethered flies produced "grooming" and "struggling" leg movements which were ideal to precede activation of freezing behavior but were not ideal for annotating takeoff behaviors. For giant fiber (GF) activation experiments, experimental and control flies were provided a small piece of a Kimwipe (Kimberly-Clark) to "kick" away during a takeoff. For freezing experiments, Kimwipes were provided to and then removed from flies to induce an active state if the fly appeared to be immobile. Once the fly was positioned, the smartphone camera was adjusted, and the fly was in the ideal behavioral state, a video recording and a 1.5 second delayed 300ms red light stimulation were initiated by touching the record button on the smartphone."

17. Line 224: Please describe how to quantify the freezing if the fly was not moving before the light ON.

    We made efforts to avoid this situation. Flies that were observed to not be moving were made active by providing them with and then taking away the Kimwipe (see above).

Response to Reviewer 2:

1. _I like the automatic triggering of the arduino with the ultrasound sensor, but it would be useful to see more details on why this was created in the "Controller" section of the paper. What level of synchronisation was achieved? In other words, using this system, would the optogenetic protocol always start at a specific time interval after the video started? What was the error? I am trying to understand if this sync system is crucial for this setup, or if it is more of a "nice to have".

    We appreciate that this gap in the description was caught, and we have added the missing details (Lines 610-619), as the synchronization between the camera and LED is crucial. The error associated with timing in Arduino Uno R3 modules was found to be +/- 0.003% which is based on the frequency tolerance (30 ppm) of the specific crystal used

for timing (taken from the Arduino Uno R3 datasheet and HC-49USX Quartz Crystal datasheet). For the 1.5 second delay (from camera activation to light stimulation) this creates a negligible +/- 45 microsecond variability.

"When the Motion Sensor Module detected a finger press of the smartphone camera shutter, the RGB LED in the Controller LED Module changed from green to blue. After a 1.5 second delay, the level of digital pin D7 was changed from LOW to HIGH for 300 milliseconds, causing a 300-millisecond activation stimulation from the red LED (measured to be 0.16 mW/mm^2 at the fly's location, 2mm away from the LED). At the end of the LED stimulation, the device entered a 30 second refractory period. During this period, students would stop their video recording and the Optogenetic Stimulation LED could not be re-activated, preventing students from over-stimulating their fruit fly and providing enough time for CsChrimson to recover from deactivation [46]. The timing of the video capture and light stimulation enabled students to consistently collect fly behavior before, during and after the light stimulation."

2. **Add presentation of raw data in Figure 4 (strong recommendation).** Figure 4. I get the effect of the workshops is significant, but I would like to see the raw data plotted here. This would allow for a better insight on how the student's prior level of knowledge is spread, and how less spread it becomes after the workshops. If I understood correctly you have ~45 students in total, and there is no obvious reason (at least not described on the text) to split the data into workshop groups. So it would be possible to simply plot all the data points, and indicate where means and percentiles are (something similar to this)

   Agreed, we have updated this figure (See Reviewer 1, comment 2)

3. I like the rationale put in the introduction and how it breaks down things step by step. The one shortcome, is that it fails to mention other open source optogenetics systems and make a brief comparison with the one described here. A brief pubmed search brought up these resources (1, 2, 3,4), maybe they are pertinent for the paper/comparison? (from what I see, the device described here is the most affordable one).

   We have added missing references and agree that they are pertinent for the paper. We have added the references and updated our introduction (See Reviewer 1, comment 5).

4. Another general point is that for this system to work, users need to have access to a smartphone (I understand that we normally think most people have one, but this is not necessarily true for high school students outside US, Europe, and certain regions in Asia), and a stereoscope (this is def. Not always the case in all high schools). If the authors could add a couple of words on alternative ways to still perform the experiments using other resources, I think this would help readers interested in bringing this to their classrooms.

   We have addressed this issue in our discussion. We believe the smartphone is still the most affordable accessible option (in comparison with high-speed cameras) but do

believe we can design a future device that eliminates the need for a stereoscope (Lines 1045-1052).

"Our device also requires a functioning stereoscope which may not be present in all high school labs. Future iterations of the device could incorporate a smartphone camera mount, 3-axis-manipulator, and a smartphone macro lens to eliminate the need for a stereoscope. Finally, our workshop assumes at least one high school student in every group of three will have a functioning smartphone. We incorporated smartphones into our device as they are currently the most ubiquitous technology for videography. To overcome financial barriers, however, smartphones could be replaced by high-speed cameras as the cost of the technology decreases."

5. Line 67 Could the authors expand a bit the phrase relating to optogenetics being predicted to be less invasive for human brain stimulation? It is less invasive in comparison to what, and in what sense? My rationale is that for human brain stimulation you would need to genetically alter brain regions to express specific proteins and depending on areas to be stimulated, one would still need to insert a fibre/fibres.. Which makes "invasive" a very relative term?

   We agree that any kind of optogenetics therapy for brain stimulation would require some kind of gene therapy and some kind of implantable medical device, like an optical window over the cortex, which is invasive. However, we would argue that this is less invasive than the current approved technology (Utah array, deep brain stimulators) where electrodes are directly implanted into brain tissue. We have clarified this in the introduction and added relevant references (Lines 80-91).

   "Optogenetics has been fundamental for recent advances in mapping functional brain connectivity [28, 29], and may emerge as a less invasive, neural cell-type specific option for human brain stimulation, as compared to current electrode-based cortical and deep brain stimulators [30-36]. "

6. Line 204 is the phone app only available for Iphone? If this is the case, please add alternatives for Android phones. If that is not available, please state it in the text.

   We have made this clarification and discuss alternatives for Android phones (see Reviewer 1, comment 15)

7. Line 244 Please add a link to the summer academy program where the workshops take place.

   This is a great suggestion, we have added the link (Line 776)

   "https://drexel.edu/biomed/resources/prospective-students/summer-academy/"

8. Please add links to the materials needed. I know you write the name of the kits and the red LED, butadding the links will help readers procure them. If they have Amazon available, this will make ordering faster, and if not, they will be able to at least see what they look like and find similar products.

*Thank you for this suggestion, we have compiled a supporting materials table for the methods section (**S1 Table. Materials with purchasing information)** and have added links to all non-generic materials. We have also included this table in our protocols.io.*

9. If possible, add a pdf version (or other available file format) of the protocols.io page to your github repository, so that there is at least one place all needed protocol information is available.

   This was a great suggestion. We have added the PDF of the protocols.io page to our GitHub repository.

10. Giant Fiber abbreviation is introduced twice, once in line 216 and once in line 311. Please correct.

    We made the correction to introduce the abbreviation once.

11. On your protocols.io description, please add photos or a video showing step 13, as this is a crucial step of the preparation.

    We agree and have now added a tethering figure to our protocols.io.

12. Line 316 two spaces after the full stop

    Edited to be only one space.

13. Line 318 space between word and full stop

    Edited to remove the space.

14. Line 339 “labled”

    Spelling has been corrected.

Response to Reviewer 3:

1. <u>Alternative to UV glue in high schools where UV light sources might not be available (strong recommendation).</u> Line 195: Use of UV glue necessitates access to a directable UV light source, which could be problematic for high school teachers. Suggest commenting on possible alternatives methods.

   Thank you for pointing this potential limitation. We have tested alternatives to the UV glue and powerful UV light (>$200) we commonly use in the lab: an inexpensive light curing glue and rapidly drying glues. We do not recommend the use of rapidly drying glues, in our hands they were less viscous and doused the flies and did not cure prior to flies recovering from cold anesthetization. We recommend the use of an inexpensive

light curing glue (listed as Liquid Plastic Adhesive Kit on the S1 Table). The Liquid Plastic glue was viscous enough to stay in place on the fly when tethering, and the light source provided by the inexpensive kit (<$20) cured the glue within 5 seconds of light exposure. Although the product did not contain an eye protection warning, we still recommend the use of UV goggles when using this product (listed in our protocols.io safety and warnings section).

2. The authors make strong claims about the novelty of bringing inexpensive tools for Drosophila optogenetics and neurotechnology more generally into classrooms. There have been quite a few efforts to do this and the authors could do a better job of acknowledging this body of work and then soften some of their claims accordingly.

   In particular, there have been quite a few publications led by academics that outline the use of Drosophila neurogenetics and inexpensive tools for optogenetics in classrooms. There have also been strong efforts to bring neurotechnology in the form of neurophysiology into classrooms led by Backyard Brains. It would be nice to see sections added into the introduction and discussion that comment on how the work presented here extends beyond these efforts into new domains. The use of motion sensors to trigger LEDs and the use of a 'Slomo' app for high-speed videography for example, opens up fantastic opportunities for studying fast behaviours. Below is a list of articles that I would suggest reviewing and considering for citation at various points in introduction and/or discussion.

   • Pulver et al., 2011 (first report of inexpensive Drosophila optogenetics methods in classrooms) https://journals.physiology.org/doi/full/10.1152/advan.00125.2010
   • Marzullo et al., 2012 (presentation of inexpensive amplifier now widely used in education https://www.ncbi.nlm.nih.gov/pmc/articles/PMC3310049/
   • Pulver and Berni, 2012 (reviews of use of Drosophila neurogenetics in teaching labs) https://www.ncbi.nlm.nih.gov/pmc/articles/PMC3592735/
   • Titlow et al., 2014a,b (reports of deployment of Drosophila optogenetics in high schools) https://uknowledge.uky.edu/cgi/viewcontent.cgi?article=1081&context=biology_facpub and https://f1000research.com/articles/6-117/v1
   • Michels et al., 2017 (multi-lingual resources for deploying Drosophila neurogenetics in secondary school classrooms) https://www.ncbi.nlm.nih.gov/pmc/articles/PMC5395560/
   • Chagas et al., 2017 (inexpensive lab for optogenetics) https://www.ncbi.nlm.nih.gov/pmc/articles/PMC5515398/
   • Rhodes, unpublished (online SFN training resources for educators) https://neuronline.sfn.org/training/module-5-implementing-optogenetics-in-the-classroom
   • Villinsky et al., 2018 (refinement of optogenetics exercises, including inexpensive methods for activation of giant fiber system in adult flies) https://pubmed.ncbi.nlm.nih.gov/30254546/
   • Tadres and Louis, 2020 (inexpensive system for closed loop optogenetics experiments) https://journals.plos.org/plosbiology/article?id=10.1371/journal.pbio.3000712

   This is not meant to be an complete list of all relevant work; there are certainly other sources that could be cited. Just to be clear, in this type of report, I would of course, not expect an exhaustive review of the entire field, but think it is appropriate to include a few lines acknowledging efforts to make specific relevant types of neurotechnologies more accessible to educators, as this is a core theme of the paper

We thank you for these references and now mention them in the introduction (see Reviewer 1, comment 5)

3. Title: Suggest modulating to emphasize use of high speed videography to distinguish this work from other attempts to bring optogenetics and neurotechnology generally into classrooms.

   Thank you, we have followed your advice and edited our title:

   "An optogenetics device with smartphone video capture to introduce neurotechnology and systems neuroscience to high school students"

4. Abstract: The abstract makes a very strong initial claim to the effect that neurotechnology is not being brought into high school classrooms, when in fact there are quite a few ongoing efforts to do this (see comments on intro and discussion). Suggest softening this initial claim to be inclusive of ongoing efforts to bring neuroscience technologies into classrooms. Bringing cutting edge neuroscience technologies into high school classrooms can be perhaps be presented as an ongoing challenge rather than as a current 'failure to follow suit'.

   Abstract: Suggest acknowledging that Drosophila optogenetics is now beginning to be deployed in both undergraduate and high school classrooms, but then pointing out the lack of tools for quantitatively studying high speed behaviours. This would then provide a nice rationale and underscore the specific value of this study.

   Abstract: The type of optogenetics experiments performed are not well defined in the abstract. Suggest modulating abstract to report on the actual experiments done (i.e. fast escape behaviours in adult flies). This will help readers understand the scope of the experiments presented.

   We have incorporated these comments and edited the abstract as follows (Lines 27-40)

   "Although neurotechnology careers are on the rise, and neuroscience curriculums have significantly grown at the undergraduate and graduate levels, increasing neuroscience and neurotechnology exposure in high school curricula has been an ongoing challenge. This is due, in part, to difficulties in converting cutting-edge neuroscience research into hands-on activities that are accessible for high school students and affordable for high school educators. Here, we describe and characterize a low-cost, easy-to-construct device to enable students to record rapid *Drosophila melanogaster* (fruit fly) behaviors during optogenetics experiments. The device is generated from inexpensive Arduino kits and utilizes a smartphone for video capture, making it easy to adopt in a standard biology laboratory. We validate this device is capable of replicating optogenetics experiments performed with more sophisticated setups at leading universities and institutes. We incorporate the device into a high school neuroengineering summer workshop. We find student participation in the workshop significantly enhances their understanding of key neuroscience and neurotechnology concepts,

demonstrating how this device can be utilized in high school settings and undergraduate research laboratories seeking low-cost alternatives. “

5. Introduction: Line 48: The authors state:
   “In recognition of this trend, universities have significantly increased their offerings of undergraduate courses in neuroscience and neuroengineering (6). High school curricula, however, have not followed suit (7, 8).”

   Suggest softening this statement to acknowledge previous attempts to bring neurotechnology into high schools. I would suggest citing the work of Backyard Brains, Cooper lab at U. of Kentucky, and Gerber lab at the Leibniz Institute for Neurobiology, for specific examples of efforts to include neurotechnology and neurogenetics into high school curricula.

   We have softened this text (Line 60), but want to emphasize we have not found national or state level initiatives to make neuroscience a required part of high school curriculums. And we have also found high school students in our workshop have a very limited understanding (or misunderstanding) of basic neuroscience concepts. But we agree that we failed to acknowledge all of the efforts initiated outside of the public high school curriculums and have highlighted this in our introduction (Lines 73-77).

   “High school curricula, however, have lagged behind [7, 8].”

   “In recognizing this need, there have been concerted outreach efforts from higher education, the nonprofit research sector, and the private sector to develop low-cost versions of cutting-edge devices that enable high school students to employ neurotechnologies and engage in hands-on activities to learn neuroscience concepts [8, 19-27].”

6. Line 62: Suggest softening this claim to acknowledge previous work.

   Line 68: The statement ‘However, most cutting-edge neuroscience/neurotechnology experiments are costly and unrealistic to replicate in a high school setting (18).’ is not accurate. Again, suggest narrowing this claim to acknowledge previous work. Authors could make the point that although progress has been made in this domain, there is much work still to be done!

   Line 73-86: This paragraph should be rewritten to acknowledge previous efforts to build and test inexpensive systems for Drosophila optogenetics. In particular, it is not accurate to claim that there are no inexpensive systems for performing optogenetics experiments in Drosophila. There are actually quite a few available at this point (see ref list above). It is true though that there is a need for new systems, especially those that enable quantitative analysis of high speed behaviours in classrooms.

   We have added missing references and rewritten these sections (see Reviewer 1, comment 5)

7. The supplemental materials are solid, but suggest including a simple table that has sources for all components and materials needed. Suggest also reviewing methods to ensure sources of materials and reagents are documented appropriately in text.

   We agree that this is helpful and have added the table (see Reviewer 2, comment 8).

8. Line 186: 'Retinal' should be changed to 'all-trans-retinal' to reduce confusion for readers.

   We edited the text using "all-trans-retinal" at the beginning of the methods section and emphasized this in the discussion (Lines 642-644 and Lines 1001-1054)

   "*Drosophila melanogaster* were raised in the dark, in foil covered vials, on standard cornmeal/molasses medium (control flies), or as larva on standard food plus 0.2 mM all-*trans*-retinal (retinal) that was switched to standard food plus 0.4 mM retinal upon eclosion"

   "At present, the workshop requires a partnership with a *Drosophila* research laboratory in order to obtain all-*trans*-retinal fly food, as there is no current commercial source. β-carotene has been used as an alternative to all-*trans*-retinal in Drosophila optogenetics experiments [49]."

9. Line 296: Suggest delineating how exactly students annotated behaviours – did they simply review on their phones and note times of events manually?

   Agreed, a description of annotation activities was added (Lines 837-840):

   "After performing an experiment, the students then annotated the fly's behavior by reviewing the video on their phone and noting the time of escape or freezing events. Middle leg extension and flight initiation were classified as escape behaviors, and a cessation of fly motion for more than 120 ms was classified as a freezing behavior."

10. Line 317: suggest checking to see if GF driver line used was same as in Villinsky et al., 2018. If so suggest citing that paper.

    It is not the same driver line – we have a table in the methods section that lists all driver lines used and appropriately attributes them to where they were first developed/published (lines 217-218).

11. The discussion is well written, but it could be improved by addition of a section that compares and contrasts this work with other efforts to perform optogenetics experiments inexpensively, especially in adult flies. An honest presentation of the pros and cons of this approach compared to other systems would really enrich the discussion and help guide educators towards the right solution for their own particular classes.

Agreed, and we have expanded our discussion as suggested (Lines 991-1069).

"We demonstrate that our well characterized device can be successfully incorporated into a hands-on neuroengineering workshop for high school students. We find the workshop greatly improves the knowledge of all participants across four key neuroscience/neurotechnology concepts. The demonstrated device utility and discoveries made when validating the device highlight the device's adoptability in high school labs: our device is low cost, made partially of recycled cardboard, and can be assembled by a tech savvy high school student with guidance from an instructor. Our device also overcomes limitations of prior high school focused devices in that it enables the acquisition and quantification of behavioral data [27, 37].

We also highlight challenges associated with our workshop/device that may be improved upon with future renditions. At present, the workshop requires a partnership with a *Drosophila* research laboratory in order to obtain all-*trans*-retinal fly food, as there is no current commercial source. β-carotene has been used as an alternative to all-*trans*-retinal in Drosophila optogenetics experiments [49]. Adding carrot juice to fly food could therefore be a possible low-cost, easily accessible alternative but this has yet to be tested. Next, while we have found tethering under a stereoscope to be sufficient for a trained student, a newly designed, inexpensive fly tethering apparatus could be incorporated into the workshop to enable a novice to consistently tether flies [21]. Third, our device currently runs in open loop. Although this is appropriate for the behaviors we are characterizing, incorporating closed loop strategies implemented in other lower-cost devices may make our device useful in investigating a broad range of fly behaviors [20, 21]. Our device also requires a functioning stereoscope which may not be present in all high school labs. Future iterations of the device could incorporate a smartphone camera mount, 3-axis-manipulator, and a smartphone macro lens to eliminate the need for a stereoscope. Finally, our workshop assumes at least one high school student in every group of three will have a functioning smartphone. We incorporated smartphones into our device as they are currently the most ubiquitous technology for videography. To overcome financial barriers, however, smartphones could be replaced by high-speed cameras as the cost of the technology decreases. In all, we anticipate advancements in video technology will enable our device to be adopted in undergraduate and graduate research programs looking for low-cost alternatives."

12. Line 393: iPhones are quite expensive and beyond the budget of many students and teachers. Suggest commenting here explicitly on performance and suitability of less expensive phones and imaging devices.

    We have incorporated these comments into our discussion (Lines 1063-1069):

“Finally, our workshop assumes at least one high school student in every group of three will have a functioning smartphone. We incorporated smartphones into our device as they are currently the most affordable and accessible technology for our videography needs. To enhance adoptability, we plan to replace smart phones with high-speed cameras as the cost of the technology decreases. In all, we anticipate technological advancements in camera technology will enable current or future versions of our device to be adopted in undergraduate and graduate research programs looking for low-cost alternatives.”

13. Line 401: This may be a good place to comment on the challenges associated with working with tethered adult flies. Are there for example variants of these experiments that do not require UV glue and tethering?

    You can place a fly in a small cell culture dish, activate neurons, and then manually score the behavior. However, there is currently not a low-cost way to record the behavior at high speed and high resolution using this set up, because even the small culture dish enables the fly to move in and out of focus. We have a senior design team currently working on this problem, as we would love to have videos of freely behaving instead of tethered flies and be able to track all legs and wings at high resolution. In the meantime, to make tethering easier, we could perhaps adopt the setup designed by the Reiser lab for tethering flies. We comment on this in the discussion (Lines 1055-1058).

    “Next, while we have found tethering under a stereoscope to be sufficient for a trained student, a newly designed, inexpensive fly tethering apparatus could be incorporated into the workshop to enable a novice to consistently tether flies [21].”

14. Line 404: check for consistency of spelling of ‘retinal’ throughout manuscript.

    The spelling is now consistent throughout the manuscript.
